# Spin texture of time-reversal symmetry invariant surface states on W(110)

**DOI:** 10.1038/srep29394

**Published:** 2016-07-12

**Authors:** D. Kutnyakhov, S. Chernov, K. Medjanik, R. Wallauer, C. Tusche, M. Ellguth, S. A. Nepijko, M. Krivenkov, J. Braun, S. Borek, J. Minár, H. Ebert, H. J. Elmers, G. Schönhense

**Affiliations:** 1 Institut für Physik, Johannes Gutenberg-Universität Mainz, Staudingerweg 7, 55128 Mainz, Germany; 2 Max-Planck-Institut für Mikrostrukturphysik, Weinberg 2, 06120 Halle, Germany; 3 Forschungszentrum Jülich, Peter Grünberg Institut (PGI-6), 52425 Jülich, Germany; 4 Helmholtz-Zentrum Berlin für Materialien und Energie, Elektronenspeicherring BESSY II, Albert-Einstein-Str. 15, 12489 Berlin, Germany; 5 Department Chemie, Physikalische Chemie, Universität München, Butenandtstr. 5-13, 81377 München, Germany; 6 New Technologies - Research Center, University of West Bohemia, Univerzitni 8, 306 14 Pilsen, Czech Republic

## Abstract

We find in the case of W(110) previously overlooked anomalous surface states having their spin locked at right angle to their momentum using spin-resolved momentum microscopy. In addition to the well known Dirac-like surface state with Rashba spin texture near the 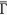-point, we observe a tilted Dirac cone with circularly shaped cross section and a Dirac crossing at 0.28 × 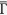 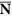 within the projected bulk band gap of tungsten. This state has eye-catching similarities to the spin-locked surface state of a topological insulator. The experiments are fortified by a one-step photoemission calculation in its density-matrix formulation.

In the past decade topological insulators have attracted large scientific interest because of their unusual electronic properties^1^,^2^,^3^. Topologically protected Dirac-type surface states appearing in the bulk band gap give rise to metallic behavior at their surfaces^2^,^3^. The rigid spin-locking of these surface states perpendicular to the crystal momentum bears high potential for the development of novel spintronic devices and improvement of existing electronic devices, suitable for spin injection and manipulation without applying external magnetic fields^3^.

It was a surprise that recently strong spin-polarized surface states with linear dispersion resembling a Dirac-cone were found on metallic surfaces^4^,^5^,^6^. This was unexpected as, e.g. W(110) has no similarities to known topological insulators except the strong spin-orbit interaction due to a large atomic number. Instead of the fundamental band gap of an insulator, tungsten exhibits a spin-orbit induced local band gap^7^. The energy range of the observed surface state is populated by *d* electrons while the fundamental band gap in known topological insulators (e.g. Bi_2_Se_3_) is caused by *p* electrons.

Miyamoto *et al*.^4^,^5^ found a “massless” (i.e., Dirac-like) surface state with linear dispersion and Rashba-type spin signature in a large energy range of 220 meV, which is an anomalous behavior in metals. Further experimental^8^,^9^,^10^,^11^,^12^ as well as theoretical work^4^,^5^,^7^,^9^,^13^ was performed in order to clarify the origin of this anomalous surface resonances on W(110). Additionally, the “direct neighbors” of W(110) in the periodic table, Mo(110) and Ta(110), were investigated. The analogous surface resonance was confirmed in our previous work for Mo(110)^14^, whereas Ta(110) does not show a “Dirac-like” surface state^15^. For Mo(110) we found a second state with anomalous dispersion behavior in the middle between the 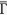 and 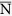 points^14^. Also, recent work of K. Miyamoto *et al*.^16^ experimentally confirmed the prediction of ref. 9, i.e. the change of spin polarization comparing p- and s-polarized light excitation. This effect is explained by an orbital-symmetry-selective excitation of states. This new work also comes to the conclusion that p-polarized light reflects at least the sign of the ground state spin polarization.

Here, we give for the first time evidence for a time-reversal symmetry invariant surface state with high spin polarization inside a spin-orbit band gap of W(110). The newly found anomalous surface state appears at 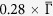 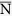 inside the projected bulk band gap. This state has striking similarities to the surface state of a topological insulator. Our experimental results also confirm the spin texture of a strongly elliptically warped surface state near the 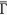 point. This band is a surface resonance hybridizing with bulk bands near the crossing point. A third anomalous band feature occurring near 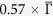

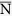
 turns out to result from a pair of spin-locked surface states, which do not have the typical cone-like appearance. These results are facilitated by time-of-flight (ToF) spin-resolved momentum microscopy allowing for a parallel detection of spin resolved three-dimensional (*k*_*x*_, *k*_*y*_, *E*_*B*_)-maps. Thus, this method allows finding spin textures off the usually studied high symmetry points that are easily overlooked by measuring with standard techniques.

## Results

### Spin-integral measurements

**Figure 1:**
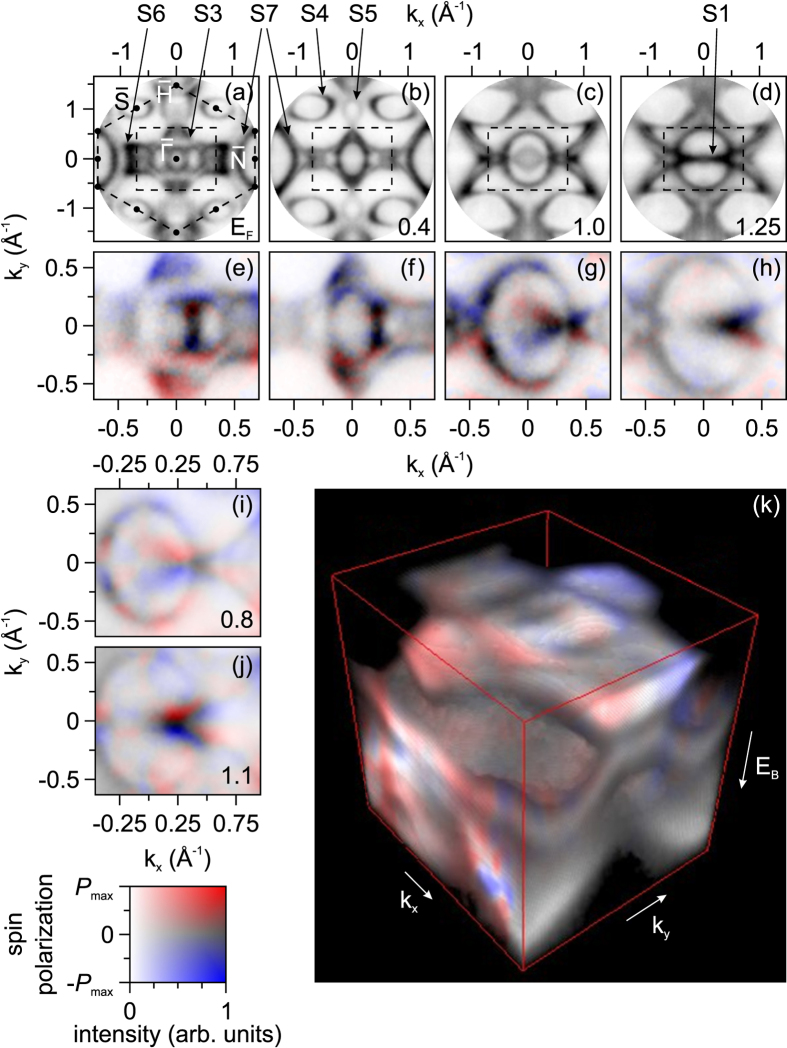
Constant-energy sections through the 3D data array. Sections represent momentum distribution images at the respective binding energy *E*_*B*_ (in eV), as indicated in the panels. Experimental momentum patterns (**a–d**) and corresponding spin-resolved images (**e–h**) taken from the areas marked by dashed rectangles. Dashed hexagon in (**a**) marks the surface Brillouin zone. Off-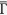 spin-resolved sections are shown in (**i,j**). Representation of the 3D array including spin information of the *E*-vs-*k* relation showing the topology of the spin resolved surface resonances on W(110) (**k**). The thin red lines define the dimensions in *k*-space being identical with the dashed rectangle as shown in (**a**) and the binding energy interval [*E*_*F*_, 3 eV]. The corresponding 2D color code is displayed in left bottom corner. *P*_max_ ≈ 0.5.

First of all, we discuss results obtained from the spin-integrated measurements of the clean W(110) surface. Figure 1(a–d) show measured constant energy sections between the Fermi energy (a) and binding energy *E*_*B*_ = 1.25 eV (d). Dashed rectangles mark areas which were measured with spin resolution as shown in (e–h) correspondingly as well as in the 3D data array *E*-vs-*k* presented in Fig. 1(k). Spin-resolved figures at *E*_*B*_ = 0.8 eV (i) and at *E*_*B*_ = 1.1 eV (j) represent details from the 3D array in order to particularly probe two new linear band crossings at *k*_*x*_ = 0.4 and 0.8 Å^−1^. In (a–d) we show only 4 out of total 100 energy slices acquired within 20 min. The data stack was treated in a way to eliminate the linear dichroism, i.e. the sections show the sum of *I*(*k*_*x*_, *k*_*y*_, *E*_*kin*_) + *I*(*k*_*x*_, −*k*_*y*_, *E*_*kin*_), making use of the mirror symmetry. Binding energies (in eV) are given at the bottom of the frames. We can clearly identify several bands. As it was already shown in previous work for Mo(110) in ref. 14 some of these correspond to surface resonances and some are bulk band features. We will see below that the agreement between experiment and theory is very good, with small deviations caused by the relative intensities of the bands and the exact positions of hybridization gaps and band maxima. The total numbers of observed and calculated bands and their principal behavior are identical. With increasing binding energy (a–d) we observe a contraction of the intense patterns S3 and S6. The oval bands around 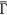 and 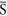 expand with increasing binding energy. The presence of the oval bands around 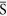 is an indication of the cleanness of the W(110) surface according to the work by Rotenberg *et al*.^17^. S7 moves towards 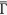 and unifies with S4, developing into a six-fold star (c, d). This star runs along the outer borderline of the bandgap as visible in (c). S5 is an oval band close to the 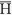 point that contracts and becomes a dot at *E*_*B*_ = 0.7–0.8 eV and forms the top and the bottom of the star as visible in (c, d) around the dashed rectangles. The anomalous band S1 only shows up in the vicinity of 1.25 eV. It is visible as a narrow ellipse at *E*_*B*_ = 1.0 eV (c), but at the crossover energy just as a single intense line running along the 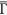-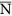 direction (d).

### Spin-resolved measurements

Spin resolved distributions were achieved by recording data sets at two working points selected by the scattering energy *E*_*Scatt*_, where highest (lowest) spin sensitivity occur at *E*_*Scatt*_ = 26.5 eV (*E*_*Scatt*_ = 30.5 eV). The potential difference between sample and spin detector was chosen such as to place the highest spin sensitivity at *E*_*B*_ = 1 eV^18^. For the data evaluation we assumed a constant value of the Sherman function *S*(*E*) = 0.2 but corrected for the energy-dependent reflectivity *R*(*E*). This assumption underestimates the evaluated spin polarization with an increasing distance from 1 eV. The resulting 3D data array of the *E*-vs-*k* spectral function with spin information is shown in Fig. 1(k). The corresponding two-dimensional color code, shown in Fig. 1 (bottom left) represents the value of the spin polarization *P* and simultaneously the intensity. Red and blue colors correspond to higher spin-up or spin-down intensities, respectively, while unpolarized intensities are shown as grey. In all cases, white means no intensity (as *P* then has no physical meaning). In comparison to spin-integrated measurements, where we examined the full surface Brillouin zone (SBZ) of W(110), the spin measurements were performed using a higher magnification of the microscope in order to look in more detail on the inner part of the SBZ.

Taking into account the given experimental geometry with p-polarized radiation in the horizontal plane preserves the mirror plane along 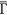

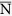
, with sensitivity to the x-component *P*_*x*_ of the spin-polarization. Artificial experimental asymmetries in the raw data were removed using the fact that the 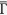

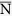
-line represents a mirror plane imposing the condition *P*_*x*_(*E*_*B*_, *k*_*x*_, *k*_*y*_) = −*P*_*x*_(*E*_*B*_, *k*_*x*_, −*k*_*y*_). As we can see from all sections (Fig. 1(e–h)), the upper (lower) half-plane with respect to the 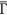

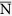
-line shows predominantly blue (red) color. Thus, e.g. at *E*_*F*_ and at a binding energy *E*_*B*_ = 0.4 eV (Fig. 1(e,f)), the spin orientation rotates clockwise for the outer (diamond shaped) and inner (oval ring) surface bands when viewed from above the sample surface. With further increase of binding energies the oval band expands. This band is a surface state centered at 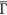. At *E*_*B*_ = 1 eV a new surface state arises close to 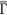 that is characterized by a high polarization opposite to the oval shaped surface band. This surface state forms the elliptically shaped Dirac cone discussed by Miyamoto *et al*.^4^,^5^ with the Dirac point at *E*_*B*_ = 1.25 eV.

**Figure 2:**
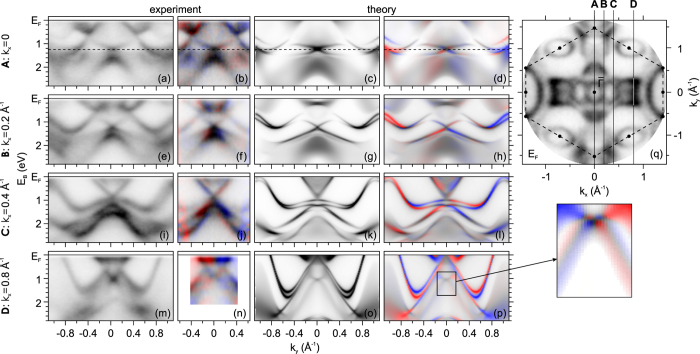
*k*_*x*_ = const. sections through the 3D data array. Sections parallel to the 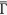 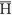-line for *k*_*x*_ = 0, 0.2, 0.4 and 0.8 Å^−1^ (1^*st*^, 2^*nd*^, 3^*rd*^ and 4^*th*^ row respectively). Col. 1, 2, 3 and 4 show experimental spin-integral and spin-resolved as well as the theoretical sections, respectively. The position of the sectional planes are denoted as **A**, **B, C** and **D** in the Fermi-surface cut (**q**). The 2D color code for spin-resolved data is displayed in Fig. 1 (bottom left).

The binding-energy dependence is visualized by extracting the band dispersions and spin-textures at *k*_*x*_ = const. parallel to the 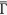 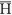-line as shown in Fig. 2(a,e,i,m) for spin-integral and corresponding spin-resolved figures (b,f,j,n). The third and fourth columns in Fig. 2 represent theoretical sections. Crossing points visible in each row at *E*_*B*_ = 1.25 eV and *k*_*x*_ = 0 Å^−1^, *E*_*B*_ = 1.25 eV and *k*_*x*_ = 0.2 Å^−1^, *E*_*B*_ = 0.84 eV and *k*_*x*_ = 0.40 Å^−1^, *E*_*B*_ = 0.96 eV and *k*_*x*_ = 0.80 Å^−1^. These sections were extracted from the 3D data array (Fig. 1(k)) along *k*_*x*_ = const. planes indicated by vertical lines **A**, **B**, **C** and **D** in Fig. 2(q). All crossing points have features of anomalous surface resonances of W(110). The linear band crossings (in Fig. 2(a–h)) correspond to the already well known Dirac-like surface state at the 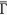 point^4^,^5^,^12^. It shows a crossing point at *E*_*B*_ = 1.25 eV with a change of the spin asymmetry and Rashba type spin texture. Two additional crossings (Fig. 2(i–p)) are new features which were overlooked earlier with downward dispersing extension due to a hybridization gap, but different topology. They show a similar reversal of the spin asymmetry from *k*_*y*_ < 0 to *k*_*y*_ > 0 close to the crossing point as present in Fig. 2(j,l) at *E*_*B*_ < 0.84 eV and *E*_*B*_ > 0.84 eV for the second Dirac-like state and in (n, p) *E*_*B*_ < 0.96 eV and *E*_*B*_ > 0.96 eV for the third one. The spin texture of the third crossing point which is situated close to 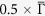 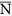 represents a quite complicated structure with spin-splitting of bands as it is seen from the theoretical section in Fig. 2(p) and magnified view (bottom right).

**Figure 3:**
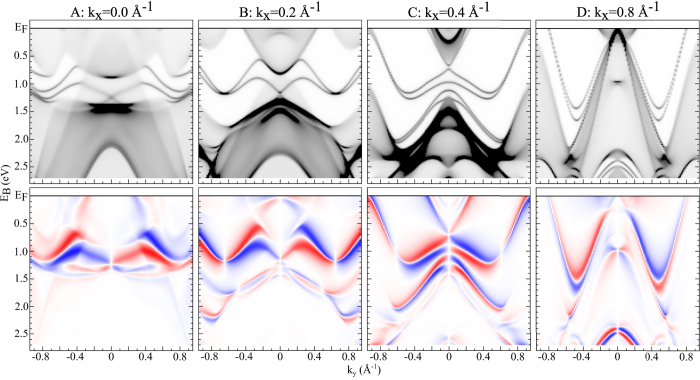
Bloch spectral functions of W(110). Theoretical spin-integral (1^*st*^ row) and spin-resolved (2^*nd*^ row) sections parallel to the 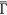 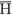 line for *k*_*x*_ = 0, 0.2, 0.4 and 0.8 Å^−1^.

For a more detailed representation of the ground state electronic structure in Fig. 3 we present Bloch spectral functions of W(110) calculated parallel to 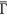 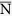-direction that represent the usual dispersion relation^19^,^20^. These calculations are based on a screened SPR-KKR formalism, where the electronic structure results for a fully relativistic self-consistent calculation for a semi-infinite stack of atomic layers. Detailed comparison of the ground state Bloch spectral functions shown in Fig. 3 and the corresponding photoemission calculations in Fig. 2 (3^*rd*^ and 4^*th*^ column) reveals that there are several changes in the spin polarization which come from the photoemission process as for example, matrix element and final state effects. This concerns in particular surface states that disperse in the band gaps and are located at higher *k*_*y*_ values (~0.6–0.8 Å^−1^). On the other hand, the spin polarization of spin-locked surface states close to the Dirac-cones discussed in this article shows the same polarization pattern as the ground state. Following our previous very detailed theoretical analysis^7^, it was shown that the spin polarization of the Dirac like state follows the ground state spin texture for a very wide range of photon energies with p-polarization (from UV up to soft X-ray).

**Figure 4:**
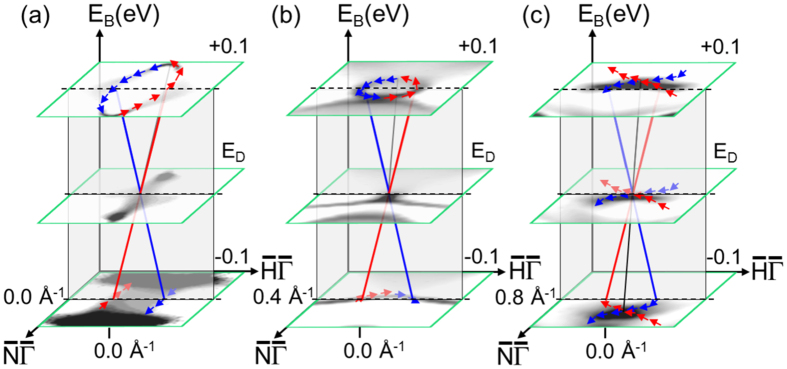
Topology of linear band crossings. (**a**) Sketch of spin-locked surface state near the 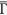 point with Dirac point at *E*_*D*_ = 1.25 eV. Horizontal planes indicate calculated constant energy slices at *E*_*D*_, and *E*_*D*_ ± 0.1 eV. The perpendicular plane displays the linear *E (k*_*x*_ = 0, *k*_*y*_) dispersion with opposite spin orientation. (**b,c**) similar data near the Dirac point at (*k*_*x*_ = 0.40 Å^−1^, *k*_*y*_ = 0.0 Å^−1^, *E*_*D*_ = 0.84 eV) and the linear band crossing at (*k*_*x*_ = 0.80 Å^−1^, *k*_*y*_ = 0.0 Å^−1^, *E*_*D*_ = 0.84 eV). Blue and red colors represent directions of in-plane spin orientation extracted from the experimental and theoretical data shown in Fig. 1(e–h,j) and in Fig. 2 (second and fourth columns).

The topologies of the linear band crossings are sketched in Fig. 4. This figure reveals the spin texture near the three band crossings appearing in the *I*(*k*_*x*_ = const., *k*_*y*_, *E*_*B*_) maps. Near 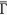 the observed spin texture confirms the previously described elliptical Dirac cone with pseudo-topological spin orientation^4^,^5^. A similar surface state was also analyzed in detail for the case of Mo(110)^14^. This state is a surface resonance as it lies at the crossing point in the region of bulk bands.

The spin texture of the band crossing near *k*_*x*_ = 0.40 Å^−1^ reveals a cone with almost circularly-shaped cross section. Its spin texture is identical with that of the surface band of a topological insulator. The tip of the cone indicates the Dirac point at (*k*_*x*_ = 0.40 Å^−1^, *k*_*y*_ = 0.0 Å^−1^, *E*_*D*_ = 0.84 eV). Between *E*_*B*_ = 0.6 eV and *E*_*D*_ this state lies completely within the projected bulk band gap^7^. Locally it fulfills all conditions for a time-reversal symmetry invariant (topological) surface state with linear dispersion near the Dirac point. Slightly below the Dirac point this cone state is crossed by a different surface band dispersing away from the 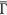 point with increasing binding energy.

The linear band dispersion observed near *k*_*x*_ = 0.80 Å^−1^ (Fig. 2 (bottom row)) is caused by two intersecting concavely shaped surface bands with clockwise and counter-clockwise spin locking, dispersing in the direction of 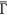. In the theoretical calculation this particular spin structure appears more complicated. The calculation (Fig. 2(p)) indicates that each of these surface bands is spin-orbit split resulting in a double cross, that is not observed in our experiment.

## Discussion

In summary, we have shown for the first time that in a projected bulk band gap of W(110) a time-reversal symmetry invariant helical Dirac state exists. This result was achieved using the novel technique of time-of-flight momentum microscopy with a W(001) imaging spin-filter. We observed a topological surface state with circularly shaped constant energy cross section and a Dirac point at 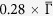 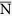 within the projected bulk band gap of W(110). This state has eye-catching similarities to the spin-locked surface states of topological insulators, the well known Dirac-like surface states with Rashba spin texture near 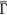. The band crossing at 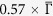

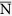
 has a more complicated structure and does not reveal Dirac-like behavior. 3D (*k*_*x*_, *k*_*y*_, *E*_*B*_)-maps in the full surface Brillouin zone with 3.4 Å^−1^ diameter and 4 eV binding energy range were measured simultaneously, resolving 2.5 × 10^5^ voxels in the spin-integral branch and more than 10^4^ voxels in the spin-resolved branch. The detailed spin texture and topology of three new band crossings with linear dispersion over a large energy range were discussed in comparison to one-step model photoemission calculations. Near 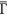 the occurrence of the elliptically shaped Dirac-type pseudo-topological surface state is confirmed^4^,^5^.

## Methods

### Experimental technique

As an experimental technique we used time-of-flight (ToF) momentum microscopy. The momentum microscope images the transversal momentum component of the photoemitted electrons on a spatially resolving detector^21^, which is combined with the ToF technique for parallel recording of electrons with different kinetic energies^22^. This gives us the (*k*_*x*_, *k*_*y*_, *E*_*B*_)-voxels of the data array which in *k*-space exceeds the first SBZ.

**Figure 5:**
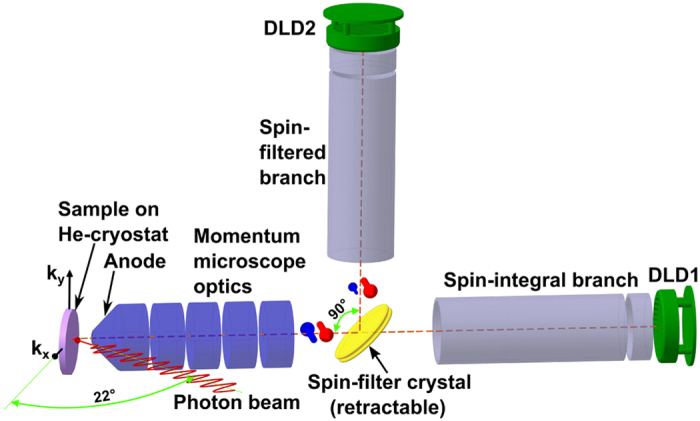
Experimental set-up. Schematic view of the spin-filtered ToF momentum microscope, consisting of He-cooled sample stage, imaging electron optics, two drift sections with delay-line detectors for spin-integral (horizontal branch, DLD 1) and spin-filtered (vertical branch, DLD 2) imaging. The W(001) spin-filter crystal is located in a field free space and can be inserted and retracted.

Figure 5 shows a schematic view of the experimental set-up. The sample is mounted on a He-cooled sample stage, consisting of a commercial helium flow cryostat and a high-precision hexapod manipulator providing a minimum temperature of 29 K (measured by a silicon diode attached to the sample holder) and six degrees of freedom for sample alignment. The imaging electron optics is the same as described previously^21^. Behind the scattering crystal are two drift sections with delay-line detectors (DLD) for spin-integral (horizontal branch, DLD 1) and spin-filtered (vertical branch, DLD 2) imaging. More details of this instrument are given in ref. 22 and the experimental geometry as well as the scheme of the 3D data acquisition is described in our previous work by Chernov *et al*.^14^. Spin-resolved images are obtained by inserting the W(001) spin-filter crystal under 45° into the electron optical path of the microscope between column and the spin-resolved ToF branch^23^. Spin contrast appears due to the spin dependent reflectivity of low-energy electrons at the scattering target caused by spin-orbit interaction at the non-magnetic surface. For the evaluation of the spin polarization we followed the recipe described in ref. 18. It requires the aquisition of two data-sets at two different scattering energies (efficient working points for a clean W(001) spin-filter): at *E*_*Scatt*_ = 26.5 eV with a reflection asymmetry of *A* = 0.3 and at 30.5 eV where the asymmetry is negligibly small^24^.

The photoemission time-of-flight experiment has been performed exploiting the time structure of the synchrotron radiation at BESSY II (Helmholtz-Zentrum Berlin, Germany) at beamline U125/2-10m Normal Incidence Monochromator (NIM)^25^ during single-bunch operation (pulse duration 50 ps, repetition rate 1.25 MHz). The monochromator provides photons in the energy range 4–35 eV and an energy resolution down to 1 meV. Given the work function of W(110) we end up with a kinetic energy range of 18 to 23 eV with the lowest 10 eV being cut off by a transfer lens^26^. The angle of incidence was 68° with respect to the surface normal, the plane of incidence was parallel to the 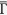 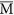 direction; the photon beam was p-polarized with the electric field vector *E* in the plane of incidence. The overall energy and *k*_||_ resolution for the present experiment were 86 meV and 0.03 Å^−1^ (best resolution of the instrument is 20 meV and 0.01 Å^−1^). For the spin-resolved measurements in the upper branch we exploited specular reflection from a W(001) spin-filter crystal at 45°. Prior to the measurements both crystals (sample W(110) and spin-filter W(001)) were treated by a standard procedure as described in ref. 24. All measurements were conducted with the sample cooled by liquid helium to 29 K.

### Theoretical approach

The calculations were done by a fully relativistic one-step model in its spin-density matrix formulation. This approach allows describing properly the complete spin-polarization vector in particular for Rashba systems using one step model of photoemission as implemented in SPR-KKR package^19^,^20^. More details of the computational method applied for the calculation of Dirac-like surface resonances on W(110) at different polarizations and photon energies are described in ref. 7.

## Additional Information

**How to cite this article**: Kutnyakhov, D. *et al*. Spin texture of time-reversal symmetry invariant surface states on W(110). *Sci. Rep.* **6**, 29394; doi: 10.1038/srep29394 (2016).
